# High Strength X3NiCoMoTi 18-9-5 Maraging Steel Prepared by Selective Laser Melting from Atomized Powder

**DOI:** 10.3390/ma12244174

**Published:** 2019-12-12

**Authors:** Angelina Strakosova, Jiří Kubásek, Alena Michalcová, Filip Průša, Dalibor Vojtěch, Drahomír Dvorský

**Affiliations:** Department of Metals and Corrosion Engineering, Faculty of Chemical Technology, University of Chemistry and Technology, Technická 5, Praha 6—Dejvice, 166 28 Prague, Czech Republic; Jiri.Kubasek@vscht.cz (J.K.); Alena.Michalcova@vscht.cz (A.M.); Filip.Prusa@vscht.cz (F.P.); Drahomir.Dvorsky@vscht.cz (D.V.); dalibor.vojtech@vscht.cz (D.D.)

**Keywords:** maraging steel, atomized powder, selective laser melting, heat treatment, precipitation hardening

## Abstract

Maraging steels are generally characterized by excellent mechanical properties, which make them ideal for various industrial applications. The application field can be further extended by using selective laser melting (SLM) for additive manufacturing of shape complicated products. However, the final mechanical properties are strongly related to the microstructure conditions. The present work studies the effect of heat treatment on the microstructure and mechanical properties of 3D printed samples prepared from powder of high-strength X3NiCoMoTi 18-9-5 maraging steel. It was found that the as-printed material had quite low mechanical properties. After sufficient heat treatment, the hardness of the material increased from 350 to 620 HV0.1 and the tensile yield strength increased from 1000 MPa up to 2000 MPa. In addition, 3% ductility was maintained. This behavior was primarily affected by strong precipitation during processing.

## 1. Introduction

Selective laser melting (SLM) is one of the best-known methods of additive production of materials. In everyday life, the most commonly encountered name for this method is 3D printing [1]. The aim of this method is to produce a three-dimensional metal part. The principle of manufacturing these parts lies in the use of a laser for melting metallic powders and subsequent layer-by-layer application of these powders. Due to the high cooling rate, rapid solidification and material transformations occur in the material. The advantage of this method is that the product is already made into the desired shape and size without the necessity for secondary machining [1]. The most useful applications of SLM technology are found in medicine, aviation, the automotive industry, and also in the work of architects [2]. 

The main advantages of 3D printing over conventional methods of manufacturing metallic materials are as follows. First, it provides the ability to produce components with various complex surface and volume shapes. This means that details can be produced during a single operation. Secondly, it requires minimal surface machining of the finished product and thus minimizes waste [1]. Third, SLM technology allows us to work with a wide range of materials. The most extensively studied are steels, Al alloys, Ti alloys, and Ni superalloys [1,3]. The microstructure of 3D printed material is almost non-porous, reaching up to 99.9% of theoretical density [1]. It is also very different from the microstructure of the materials produced by other methods. Thanks to the high cooling rate and solidification of the alloy during the SLM process, we get a material with a very fine-grained cell microstructure. Grain size reduction has a beneficial effect on the mechanical properties of 3D printed products [4,5,6,7,8]. 

Maraging steels are widely used in the aerospace industry. Also, they are widely used in tooling applications and in the production of weapons. Maraging steels are also well-known thanks to their excellent mechanical properties. The most important properties are very high hardness, good weldability, high ductility, and easy machinability after solution annealing. Spatial stability during aging is one more positive feature of the maraging steels [8,9,10,11,12,13]. Their properties and range of applications are one of the reasons for the interest in their production by using modern technologies, including 3D printing. 

Maraging steels exert these very good mechanical properties after the application of heat treatment involving solution annealing and aging. The first state after quenching is the martensitic structure of the material. This structure may be caused by different means. In maraging steels, the creation of martensite is supported by the high Ni content in an alloy. In contrast to this, in other steels, the martensitic structure is due to a relatively high carbon content. Aging at a temperature between 480 °C to 510 °C causes intermetallic precipitation [13,14]. Several phases were identified in maraging steels like Ni_3_(Ti, Mo) [13], Ni_3_X (X = Ti, Al, Mo) [10], (Fe, Ni, Co)_3_(Ti, Mo), (Fe, Ni, Co)_3_(Mo, Ti), and (Fe, Ni, Co)_7_Mo_6_ [15]. 

Several major works have been carried out to get a better idea of the properties of X3NiCoMoTi 18-9-5 maraging steel made by 3D printing. Kempen et al. [1] investigated how impact and tensile properties depend on aging treatment and process parameters. Tensile, fracture, and fatigue crack growth of maraging steel was studied by Suryawanshi et al. [4]. Some researchers [7,16] have investigated the effect of different heat treatments on microstructure and mechanical properties. In all the work mentioned above, the aging treatment significantly improved the properties due to the formation of precipitates. However, only limited information is known about the precipitates which form during the treatment of 3D printed steel. For example, the formation of a Ni3X (X = Ti, Al, Mo) precipitates after heat treatment was described by references [10,15].

The aim of this work is to investigate the impact of the 3D printing process and two heat treatment modes on microstructure and mechanical properties X3NiCoMoTi 18-9-5 maraging steel.

## 2. Materials and Methods

**Figure 1:**
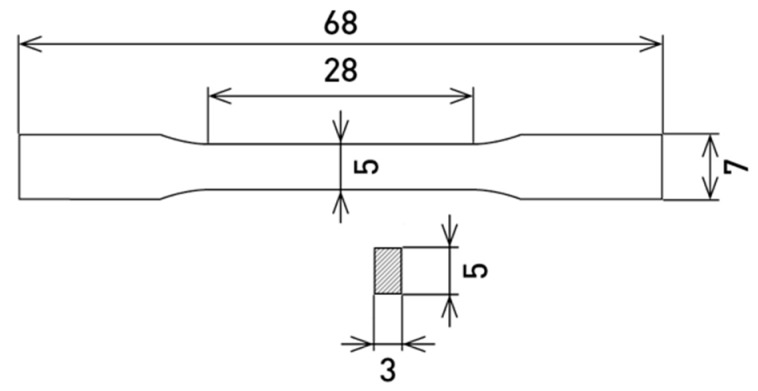
Dimensions of 3D-printed sample for a tensile test.

Samples in the shape of a dog bone (Figure 1) were produced by 3D printing on SLM solution 280HL (NETME Center, Brno, Czech Republic) from the powder X3NiCoMoTi 18-9-5 alloy. The chemical composition (Table 1) was found using XRF analysis on spectrometer ARL 9400. 

**Figure 2:**
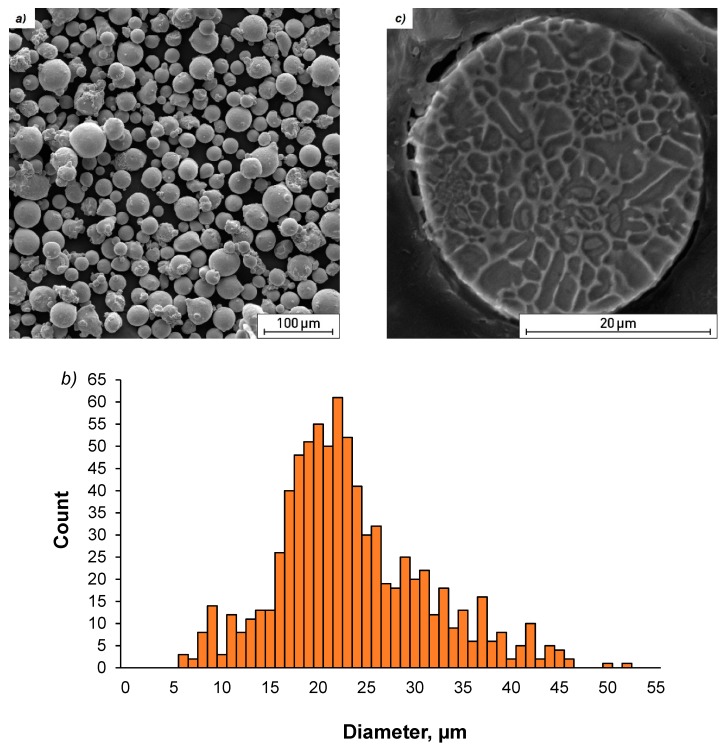
Characterization of X3NiCoMoTi 18-9-5 alloy powder; (**a**) powder morphology, (**b**) particle size distribution, (**c**) the microstructure of the powder.

The powder morphology structure and size distribution are shown in Figure 2. Particle size analysis was performed using a scanning electron microscope (TESCAN VEGA 3 LMU, Brno, Czech Republic) and the ImageJ program. 

Two heat treatment regimes were used for 3D printed specimens. The first regime was solution annealing at 820 °C for 1 h, air cooling, and subsequent aging at 490 °C for 6 h. The second one included only aging at 490 °C for 6 h. Electric resistance furnaces were used for heat treatment.

Metallographic samples were made by grinding on SiC abrasive papers with grit size P280-P4000. The samples were polished using a diamond paste (D 2 μm). Final polishing was done on an Eposil F suspension. Metallographic light microscope Olympus PME3 (LM) was used to observe the microstructure of the material. The samples were etched in Nital 2 solution (2 mL HNO_3_ + 98 mL ethanol) to visualize the microstructure. Scanning (TESCAN VEGA 3 LMU, Brno, Czech Republic, equipped by EDS-OXFORD Instruments, High Wycombe, UK) and transmission electron microscopy (JEOL 2200 FS equipped by EDS - OXFORD Instruments, High Wycombe, UK) technologies were used for a more detailed study of the structural components of the examined material. Samples for TEM analysis were prepared through grinding and polishing. A thin plate, that was cut from the studied sample, was thinned with SiC abrasive paper (P4000). Discs with a diameter of 3 mm were punched from this thinned plate, which were then polished. Polishing was carried out on PIPs GATAN in an Ar atmosphere with an ion gun setting of 5 keV and rotation of 3 rpm. Phase composition study was performed by X-ray diffraction on PANanalytical X’Pert PRO with Kα radiation on Co lamp (λ = 0.17929 nm) and generator setting of 40 mA and 35 kV in a 2θ range of 6–110° using a step size of 0.033° and scan step duration of 81.28 s.

The Vickers microhardness with a load of 100 g and holding time of 10 seconds was measured on a FUTURE TECH FM-700 machine. The microhardness was measured in the horizontal section and in the vertical section, of each sample. The final value was calculated from 10 measurements. A tensile strength test was performed at room temperature using the universal test machine LabTest 5.250SP1-VM (LaborTech, Opava, Czech Republic). The tensile test was performed only in the building direction of specimens and tensile yield strength corresponding to the 0.2 proof stress (TYS_0.2_), ultimate tensile strength (UTS), and elongation (A) were evaluated. 

## 3. Results and Discussion

### 3.1. Microstructure Characterization

**Figure 3:**
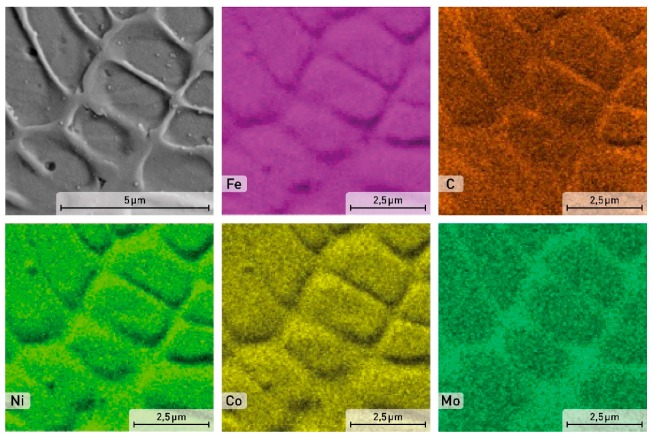
SEM image and EDS element distribution maps in the powder particle of the X3NiCoMoTi 18-9-5 maraging steel.

As can be seen from Figure 2a, the X3NiCoMoTi 18-9-5 alloy powder particles are characterized by different sizes. Most particles have almost a spherical shape that is preferred for the SLM process. Several photos of the studied powder were made using SEM and the size of eight hundred particles was calculated based on image analysis using the ImageJ software. Each particle was measured in three different directions. The particle size distribution is shown in Figure 2b. We can see that the majority of the metal particles (73%) have a size between 15 and 30 μm. The microstructure of the powder particles in the cross-section shown in Figure 2c demonstrated that the powder has a characteristic cellular structure. This cellular structure was preferred due to the high cooling rate of the powder particles during their production caused by atomization. Therefore, the alloying elements segregate at the cell boundaries (Figure 3). The phase composition of the powder of maraging steel, based on the results of XRD analysis which will be shown below, consisted of both α—martensite, and ɣ—austenite as a consequence of a rapid cooling rate.

**Figure 4:**
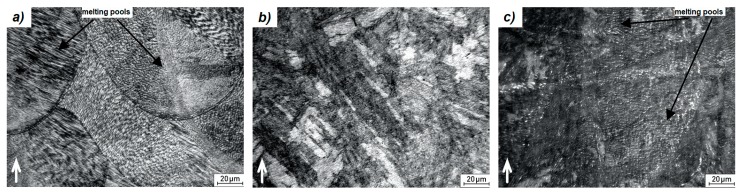
LM micrographs of the material structure; (**a**) as-printed, (**b**) solution annealed and aged, (**c**) aged; ↑—building direction.

The microstructure of the printed samples was examined before and after the application of the heat treatment regimes. As shown in Figure 4a, the sample microstructure shows the presence of very fine cellular martensite which fully corresponds to the structure of 3D printed materials as has been reported also in [1,4,10,15]. There are also visible melting pools, which show how the molten material has deposited and solidified layer-by-layer. These fine cells were formed thanks to a high cooling rate up to 10^4^–10^6^ K/s [14,16] and a difference in temperature gradients during the 3D printing.

**Figure 5:**
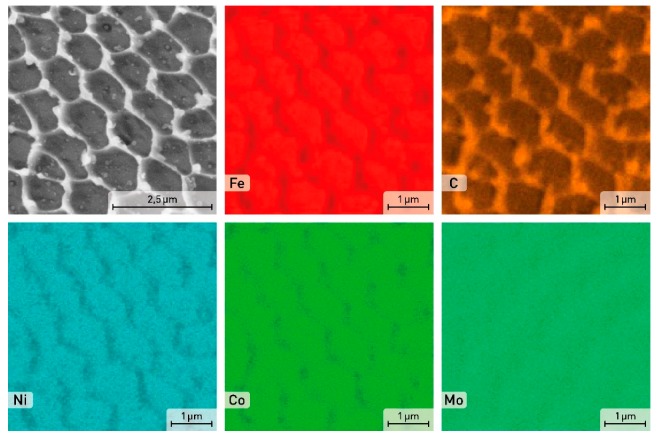
SEM image and EDS element distribution maps in the as-printed sample of the X3NiCoMoTi 18-9-5 maraging steel.

A detailed distribution of alloying elements of the as-printed sample is shown in Figure 5. The cell boundaries are enriched by C, other elements like Fe, Ni, Co, and Mo are homogeneously distributed within the cells. 

**Figure 6:**
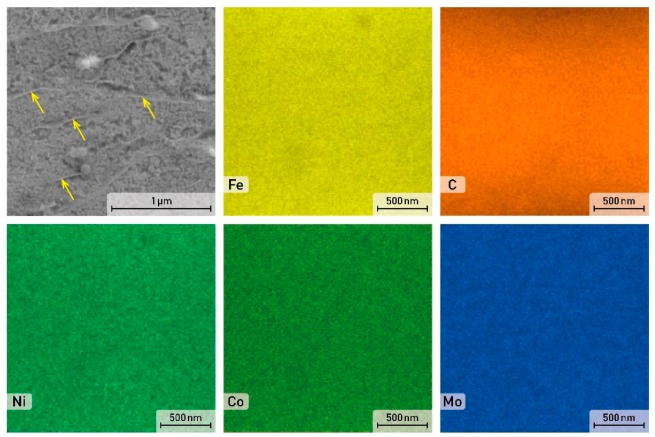
SEM image and EDS element distribution maps in the solution annealed and aged sample.

After the application of solution annealing (820 °C/1 h) and aging (490 °C/6 h), a complete change in the material microstructure can be observed (Figure 4). General characteristics of the 3D print structural components disappeared, as shown in Figure 4b. The fine-grained cell structure was transformed into a coarser needle-like martensite structure. This conversion occurred as a result of heating the material to 820 °C. At this temperature, the α-Fe solid solution became supersaturated and homogenized [10]. A more detailed view of this structure is shown in Figure 6. It can be seen that the material lost its cellular structure and was characterized by a uniform distribution of alloying elements. In addition, martensite needles are indicated by arrows in Figure 6.

**Figure 7:**
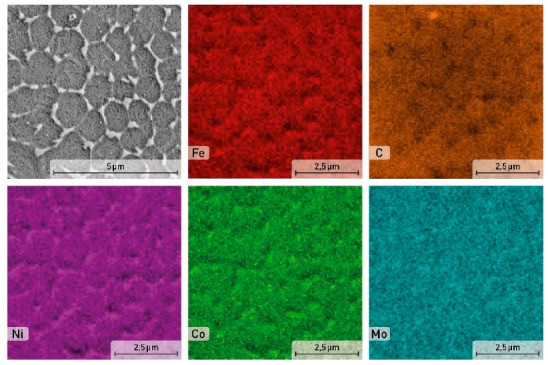
SEM image and EDS element distribution maps in the aged sample of the X3NiCoMoTi 18-9-5 maraging steel.

After aging at 490 °C for 6 h, the microstructure (Figure 4c) becomes similar to the microstructure of the as-printed sample with a specific structure. The alloying elements segregation is also located at the cell boundary (Figure 7). This phenomenon can be justified by the low aging temperature, which is not sufficient for diffusion processes to homogenize the material composition.

**Figure 8:**
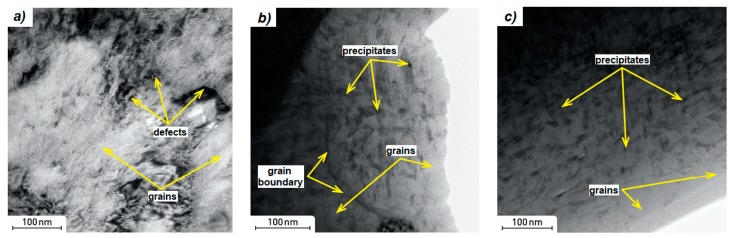
TEM images of X3NiCoMoTi 18-9-5 maraging steel; (**a**) as-printed, (**b**) solution annealed and aged, (**c**) aged.

As shown in Figure 8a, grains, grain boundaries, and also defects (mainly dislocations) are clearly visible in the microstructure of the as-printed sample by TEM. Both gas atomization process and 3D printing are characterized by high cooling rates which favor the formation of various defects like dislocations or vacancies. They also provide very high internal stresses. Based on the TEM of the as-printed X3NiCoMoTi 18-9-5 alloy, no precipitates were observed in the microstructure. The chemical composition within the cells is similar to in Table 1.

The microstructure of the heat-treated samples is different compared to the as-printed sample (Figure 8). As shown in Figure 8b,c, precipitates were formed in both solution annealed and aged samples, and only aged samples. The chemical composition of the heat-treated materials was examined using EDS analysis. Light and dark zones were analyzed with the assumption that the dark ones correspond to the precipitates (Figure 8b,c). It was found that light zones consist of 69% Fe, 15% Ni, 9.5% Co, 3% Mo and 0.7% Ti. Dark areas consist of 62% Fe, 20% Ni, 8.5% Co, 6.5% Mo, and 1% Ti. Precipitates contain a slightly higher amount of Ni, Mo, Ti, and therefore we assume the formation of Ni_3_(Ti, Mo) [10,13].

**Figure 9:**
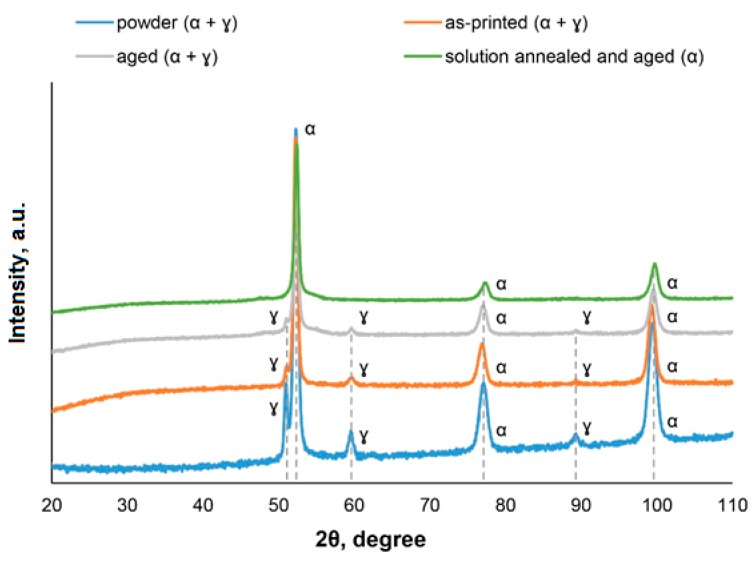
XRD patterns of X3NiCoMoTi 18-9-5 maraging steel.

The X3NiCoMoTi 18-9-5 maraging steel in four different states (powder, as-printed, aged, and solution annealed and aged was also subjected to X-ray diffraction analysis. The results are shown in Figure 9. As can be seen, the sample that has been annealed and aged contained 100% of α-phase. This confirms the martensitic structure of the material (see Figure 4b). In contrast, the retained austenite (ɣ-phase) was found in the powder, as-printed and in the aged samples. This phenomenon can be explained by methods involving studied powder or built samples. As for the aged sample, the aging temperature was not high enough to convert the retained ɣ-phase into the α-phase. According to the literature [10], the temperature necessary for this conversion is, considering the actual chemical composition, around 600 °C.

### 3.2. Mechanical Properties

**Figure 10:**
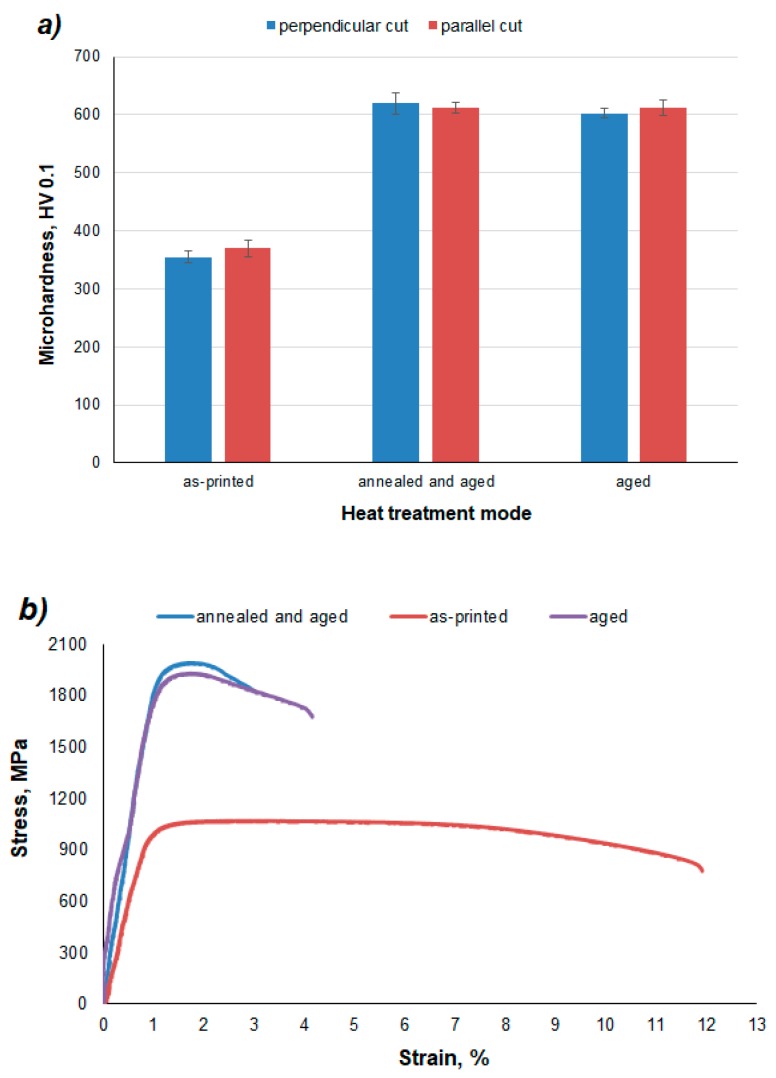
Mechanical tests of the X3NiCoMoTi 18-9-5 maraging steel depending on the heat treatment regime; (**a**) microhardness HV 0.1, (**b**) tensile test.

Figure 10 and Table 2 give us an overview of the dependence of mechanical properties on the heat treatment regimes. As can be seen in Figure 10a, the direction of hardness examination has no effect on the microhardness of the material. When either material is examined in the horizontal section or in the vertical section, the microhardness is almost the same. Thus, since the tests did not prove any anisotropic behavior with respect to the print direction, several tests were done on samples in the building direction. The hardness of the as-printed samples was relatively low. This is due to the fact that since as-printed samples have a low carbon content in martensite, martensite cannot have a really hard phase in this case.

Two regimes of heat treatment were carried out in the presented work. An interesting fact is that the resulting microhardness values were almost the same in both cases (Figure 10a). The increase in the hardness of the material after solution annealing and aging can be explained by the formation of a completely martensitic structure after solution annealing and precipitation hardening after the aging process (Figure 8b). Due to the low carbon content, martensite is not a reinforcing element. Therefore, precipitates become responsible for hardening.

After the application of aging alone, the original microstructure of the material was retained, but the temperature was sufficient to cause precipitation hardening (Figure 8c). As in the case of solution annealed and aged samples, the aged samples have a high hardness thanks to the precipitates.

Tensile tests (Figure 10b, Table 2) of 3D printed and heat-treated X3NiCoMoTi 18-9-5 maraging steel show the same trend as hardness. As can be seen in Figure 10b, the as-printed sample is characterized by low yield strength, but high ductility. In contrast, the strength characteristics of the material after heat treatment are nearly twice as high as that of the printed sample, but low ductility is observed. One can see that the annealed and aged sample has slightly higher strength characteristics, but a lower ductility compared to the sample which was only aged (Table 2). This phenomenon can be explained by phase composition (Figure 9). The aged material contains the α-phase in addition to the ɣ-phase. It is well known that the ɣ-phase is soft, tough, and ductile. In contrast, the annealed and aged alloy has a 100% α-phase and, as shown in Figure 4b, it has a martensitic structure. During the solution treatment of the as-printed sample, th cellular structure is removed and the distribution of alloying elements is homogenized. This particular leads to stronger precipitation during aging, which causes the increase of TYS and UTS, as well as the decrease of elongation.

The mechanical properties of the 3D printed maraging steel were compared with the properties of the same steel produced by conventional methods. It has been found that the hardness, ultimate tensile strength, and elongation of wrought alloys are slightly higher than of 3D printed ones [17,18,19]. This difference is justified by the presence of residual porosity in additively produced materials.

## 4. Conclusions

The effect of solution annealing (820 °C/1 h) plus aging (490 °C/6 h), and also only aging (490°C/ 6 h) on the microstructure and mechanical properties of 3D printed X3NiCoMoTi 18-9-5 maraging steel was investigated in this work. The as-printed X3NiCoMoTi 18-9-5 maraging steels were characterized by a martensitic structure with residual austenite. The material was composed of specific cells containing supersaturated solid solution and a high quantity of defects. After the application of solution annealing and aging, the cell structure disappeared and a coarse martensitic structure was formed. In addition, precipitates enriched by Ti and Mo were also formed. Aging of as-printed material caused precipitation of Ti and Mo enriched phases, but the cellular structure of the as-printed sample remained. Application of two regimes of heat treatment occurred, such as solution annealing and aging, and also only aging. The mechanical properties of the maraging steel were significantly improved after thermal processing, including annealing and aging or only aging. Hardness and tensile yield strength increased almost twice at the expense of slightly impaired ductility. Base on the observed properties, the solution annealing stage seems not to be necessary to improve the mechanical properties of 3D-printed samples. For these purposes, it is sufficient to apply only the aging regime.

## Figures and Tables

**Table 1:** Chemical composition of X3NiCoMoTi 18-9-5 maraging steel.

Element	Ni	Co	Mo	Ti	C	Al	Cr	Mn	Si	Fe
**Wt/%**	19	9.3	5	0.64	≤0.03	0.06	0.08	0.04	0.07	Bal.

**Table 2:** Summary of X3NiCoMoTi 18-9-5 alloy properties depending on heat treatment.

	A	UTS, MPa	TYS0,2, MPa
**As-printed**	11.3	1080	999
**Annealed and aged**	2.5	1992	1943
**Aged**	3.5	1944	1867
